# Cultural determinants influence assisted reproduction usage in Europe more than economic and demographic factors

**DOI:** 10.1093/humrep/dex298

**Published:** 2017-09-29

**Authors:** Patrick Präg, Melinda C Mills

**Affiliations:** 1 Department of Sociology and Nuffield College, University of Oxford, Manor Road, Oxford OX1 3UQ, UK

**Keywords:** ART, Europe, social norms, demography, ART usage

## Abstract

**STUDY QUESTION:**

To what extent do financial, demographic and cultural determinants explain the vast cross-national differences in ART treatments in Europe?

**SUMMARY ANSWER:**

The normative cultural acceptance of ART is a major driver of ART treatments in Europe, above and beyond differences in country wealth, demographic aspects and religious composition.

**WHAT IS KNOWN ALREADY:**

There are vast differences in the number of ART treatments across European countries, which are to some extent related to country affluence, regulation, and insurance coverage and costs. The role and impact of cultural and normative factors has not been explored in a larger cross-national comparison.

**STUDY DESIGN, SIZE, DURATION:**

A descriptive and comparative cross-national analysis of ART treatment prevalence in over 30 European countries in 2010, with the outcome defined as the total number of ART cycles per million women of reproductive age (15–44 years). Data is drawn from multiple sources (ICMART, US Census Bureau Library, World Bank, Barro–Lee Educational Attainment Dataset, IFFS Surveillance reports, European Values Study and World Religion Database).

**PARTICIPANTS/MATERIALS, SETTING, METHODS:**

Our sample includes data from 35 European countries, where we describe the associations between demographic and cultural factors and the prevalence of ART treatments. Bivariate correlation and ordinary least squares multiple regression analysis serves to establish the relationships between predictor variables and the number of ART treatments per million women aged 15–44 years in a country.

**MAIN RESULTS AND THE ROLE OF CHANCE:**

A one-percent increase in national GDP is associated with 382 (95% CI: 177–587) additional ART procedures per million women of reproductive age, yet this effect is reduced to 99 (−92 to 290) treatments once cultural values and demographic factors are accounted for. In our fully adjusted model, normative cultural values measuring the acceptability of ART are the strongest predictor of ART usage, with a one-point increase of average approval in a country associated with 276 (167–385) additional ART treatments per million women of reproductive age.

**LIMITATIONS, REASONS FOR CAUTION:**

Findings are based on a cross-sectional, cross-national analysis, making formal tests of causality impossible and prohibiting inferences to the individual level.

**WIDER IMPLICATIONS OF THE FINDINGS:**

Results indicate that reproductive health policy should openly acknowledge the importance of cultural norms in informally shaping and regulating the wider availability of ART treatment.

**STUDY FUNDING/COMPETING INTEREST(S):**

Funding for this project was provided by the European Union's Seventh Framework Program (FP7 2007–2013) (No. 320116 Families and Societies), European Research Council for the SOCIOGENOME Consolidator Grant (ERC-2013-CoG-615603) and the Wellcome Trust Institutional Strategic Support Fund (all to M.C.M.). The authors have no conflicts of interest to declare.

**TRIAL REGISTRATION NUMBER:**

N/A.

## Introduction

The use of ART varies considerably across countries in Europe (Calhaz-Jorge *et al.*, 2016, Präg and Mills, 2017). The most recent release of the registers of the European IVF-Monitoring Consortium (EIM) for the European Society of Human Reproduction and Embryology (ESHRE) (Calhaz-Jorge *et al.*, 2016) reveals that the number of ART treatment cycles in 2012 ranged between 1457 per million women between the ages of 15 and 44 in Moldova compared to 14 431 per million women in the same age range in Denmark. The reasons for these differences, however, are not yet fully understood.

The economic development of countries has been previously considered as a main driving factor, but cannot explain the vast differences between countries. While richer countries have a somewhat higher prevalence of ART use, the relationship is far from perfect, with many poorer countries in Europe having high levels of ART usage. For instance, the Czech Republic reported 10 473 cycles per million women of reproductive age in 2012 but ranks low (51st) in national wealth as measured by purchasing power parity GDP. The Czech Republic has an ART treatment level that is close to the comparatively wealthier Denmark (ranked 37th), whereas high-income nations such as Italy (ranked eighth) and the United Kingdom (ranked fifth) reported only 5480 and 4918 cycles per million women of reproductive age, respectively (World Bank, 2011).

Existing research points to other economic, regulatory and demographic factors underlying country differences in ART uptake. Chambers *et al.* (2014) argued that it is not only country wealth, but rather the consumer affordability of treatment that drives the country differences in ART usage. Countries where ART treatments are more affordable due to insurance mandates or public subsidies have higher ART usage, suggesting that a cost cut of 10 percentage points of the average disposable income in a country predicts a 32% increase in ART utilization. With respect to ART regulation, Berg Brigham *et al.* (2013) showed that European countries with more liberal social eligibility regulations registered higher levels of ART usage. Kocourkova *et al.* (2014) illustrate that country differences in ART usage are related to fertility postponement. The greater the extent of postponement of first birth in a country, the higher demand for ART treatments. These studies have identified important societal drivers of cross-country differences in ART utilization.

Large-scale cross-national empirical research on cultural and normative factors shaping ART usage are rare, with some studies showing that cultural factors such as social norms and religion may be important predictors of ART usage (Benagiano, 2008; Adamson, 2009). Billari *et al.* (2011) reported that country differences in social age deadlines for childbearing, such as beliefs about having a child too early or too late, were predictive of differences in ART availability in European countries.

This study examines ART utilization data from 35 European countries and examines the extent to which financial, demographic, and cultural determinants explain the vast cross-national differences in ART usage and treatments in Europe. We replicate the well-known correlation of country wealth and ART treatments. In addition, we show how demographic factors such as fertility postponement are related to ART usage. There is a well-established link between women's higher educational attainment and postponement (Mills *et al.*, 2011, Balbo *et al.*, 2013), with the proportion of higher educated women also varying across nations. We therefore focus on the group of women most likely to postpone childbearing, namely mid-aged, highly educated women. We then take regulatory aspects of ART in the countries under study into account (Präg and Mills, 2015) and make use of survey data to understand country differences in the moral and normative cultural acceptance towards ART and how religious composition of countries is related to ART usage.

## Materials and Methods

**Figure 1:**
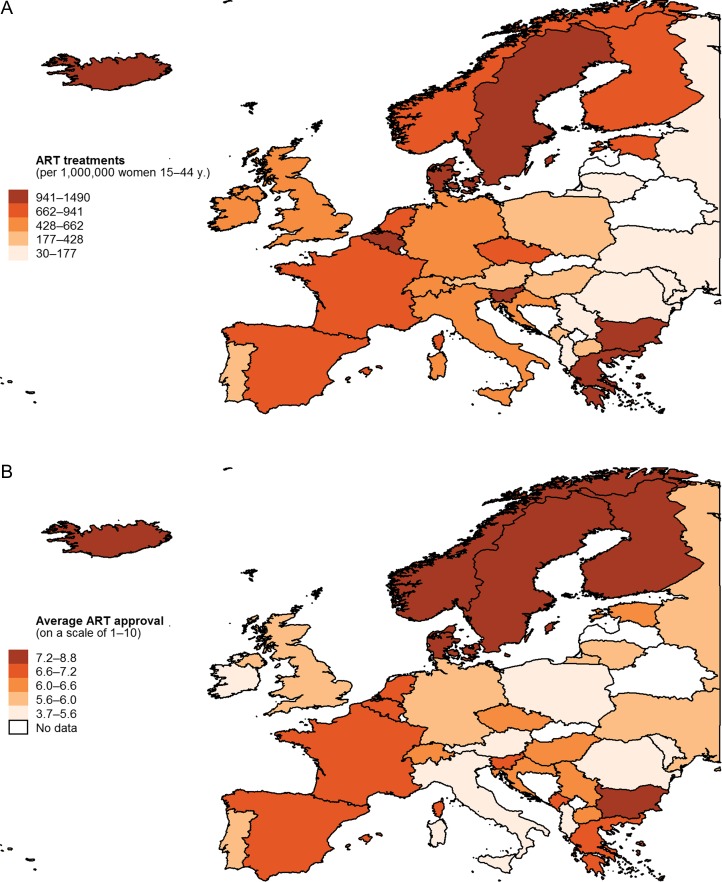
(**A**) ART treatments and (**B**) average ART approval by country, ca. 2010.

We draw on a number of different data sources for our analyses. Our outcome variable, the total number of ART cycles per million women of reproductive age (15–44 years), stems from the European countries included in the ICMART world report for 2008–2010 (Dyer *et al.*, 2016) and from the US Census Bureau Library (2016) for the number of women of reproductive age. Figure 1A shows country differences in ART usage across Europe.

We make use of a number of predictor variables that can be grouped into the categories of economic, demographic, regulatory, and cultural forces assumed to determine ART usage. As an economic predictor, GDP per capita (in purchasing power parities) was obtained from the World Bank's International Comparison Program database (World Bank, 2011). To pull in potential outliers and ease interpretation of findings, we took the natural logarithm of GDP per capita.

As a cultural predictor, we draw on the moral and normative acceptance of assisted reproduction, which is calculated in the following manner. In the 2008 European Values Study (EVS and GESIS, 2010), respondents are asked whether ‘artificial insemination or *in-vitro* fertilization’ can ‘always be justified, never be justified, or something in between’ and are presented a card with a scale from 1 to 10, where 1 is labeled ‘Never’ and 10 is labeled ‘Always’ (Szalma and Djundeva, 2017). We calculated the average response by country, where higher values denote a greater acceptance of assisted reproduction in a country. The European Values Study is a high-quality social science survey conducted every nine years, drawing on representative random sampling, large samples (~1500 respondents per country), elaborate translation processes that ensure accuracy of question translations, and extant pre-tests to ensure the validity of measures (Luijkx *et al.*, 2017). Figure 1B shows country differences in average ART approval in Europe.

**Figure 2:**
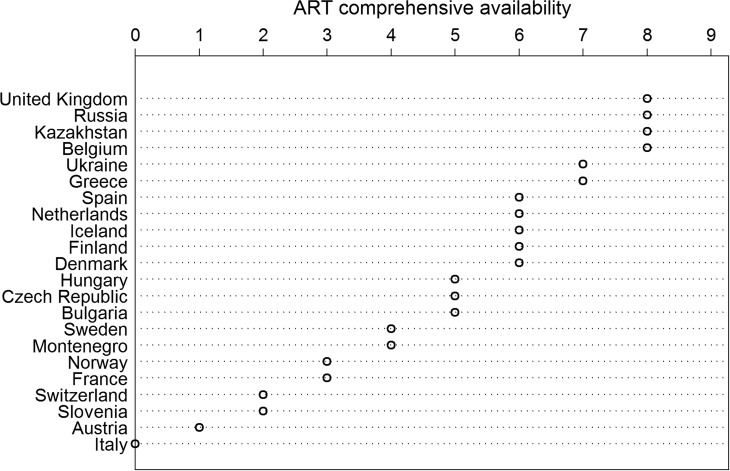
ART comprehensive ability by country, ca. 2010.

As a regulatory predictor, we calculate and introduce a new ‘ART accessibility index’ based on International Federation of Fertility Societies (IFFS) Surveillance reports (Jones *et al.*, 2007, 2010), which measures the availability of ART in a more comprehensive manner. The IFFS surveys national experts every three years on matters of ART regulation. We have standardized the reports of these experts, which served as the basis for our index (Präg and Mills, 2015). The ART accessibility index we use in this analysis is scored as follows: If ART treatment is available for: (i) single women, and, (ii) lesbian women, one point is added for each. If: (iii) sperm donation for IVF, (iv) oocyte donation, (v) embryo donation, (vi) gestational surrogacy, (vii) sex selection, (viii) posthumous insemination, and, (a) non-anonymous donation are available, one point is added for each. The resulting index ranges from 0 to 9, with a greater number indicating a more comprehensive availability of ART. While this selection of indicators is driven by data availability and might seem arbitrary or to potentially affect a relatively small part of the ART patient population directly, we believe it taps the most contentious areas of ART regulation and thereby offers an impression of the regulatory pressures that ART providers and patients are facing as well as the range of services that are available in a country. Figure 2 presents descriptive statistics of this indicator by country.

As a demographic predictor, the percentage of women with completed tertiary education between the ages of 35 and 54 is calculated based on the Barro–Lee Educational Attainment Dataset (Barro and Lee, 2013) and refers to the year 2010.

A predictor that taps both demographic as well as cultural aspects, is the population percentages of four major religious groups in European countries around 2010, namely Protestants, Catholics, Orthodox Christians and Muslims, from the World Religion Database (Johnson and Grim, 2017).

The statistical methods we rely on for our analyses are bivariate correlations and ordinary least squares (OLS) multiple regression models. We will first inspect the data in a bivariate fashion by means of scatterplots, and in a second step fit a multivariate model. In a third step, we investigated possible interactions between particularly large effects. This article is also accompanied with a replication package including all data and code in Stata format which allows readers to replicate all analyses in this study (‘replication_package.zip’ in Supplementary Information).

## Results

### National GDP wealth and birth postponement

**Figure 3:**
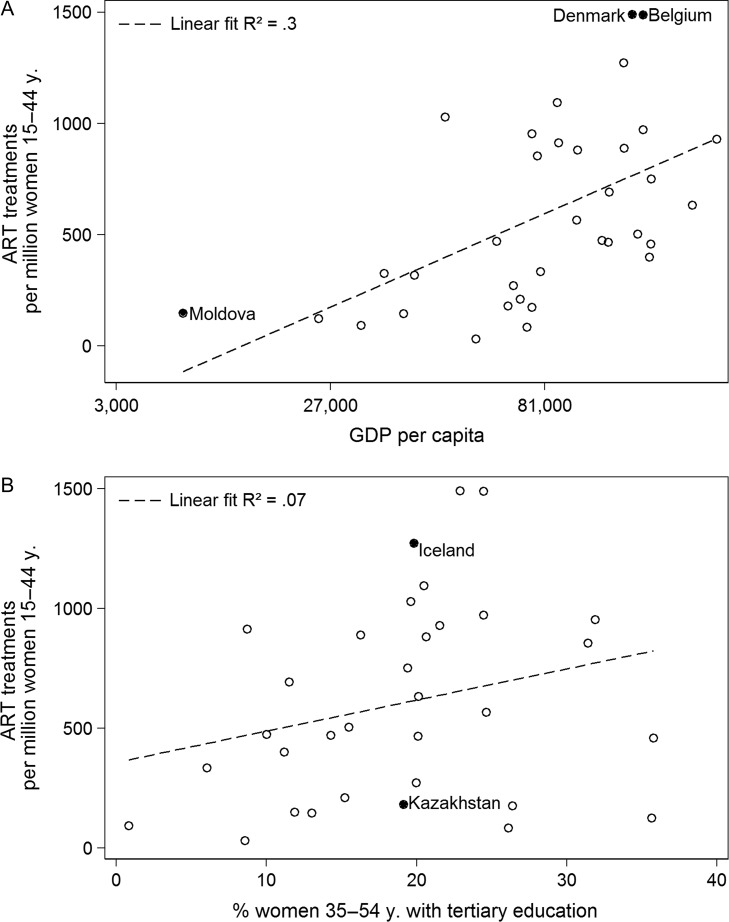
ART treatments by (**A**) GDP per capita, (**B**) % women between 35 and 54 years of age who have completed tertiary education, ca. 2010.

In line with previous findings and our observations from the introduction, Fig. 3A shows that country affluence as measured by GDP per country is an important, but not all-encompassing predictor of the number of ART treatments per million women of reproductive age. The *R*-square indicates that GDP explains 30% of the variance of ART usage between countries. Figure 3B reveals that there is indeed a relationship of ART treatments in a country with birth postponement, using the percentage of mid-aged women with higher education as a proxy indicator, but it is markedly lower (*R*-squared of 0.07) than the relationship with GDP. The average percentage of women aged 35–54 years is ~19%. Figure 3B shows that countries with approximately an average percentage of mid-age women with a tertiary educational degree range from <200 (Kazakhstan) to more than 1200 (Iceland) treatments per million women of reproductive age. This illustrates the variability of ART usage across countries around the key demographic variable of women's higher levels education, which is strongly related to birth postponement.

### Normative cultural approval of ART and the ART accessibility index

**Figure 4:**
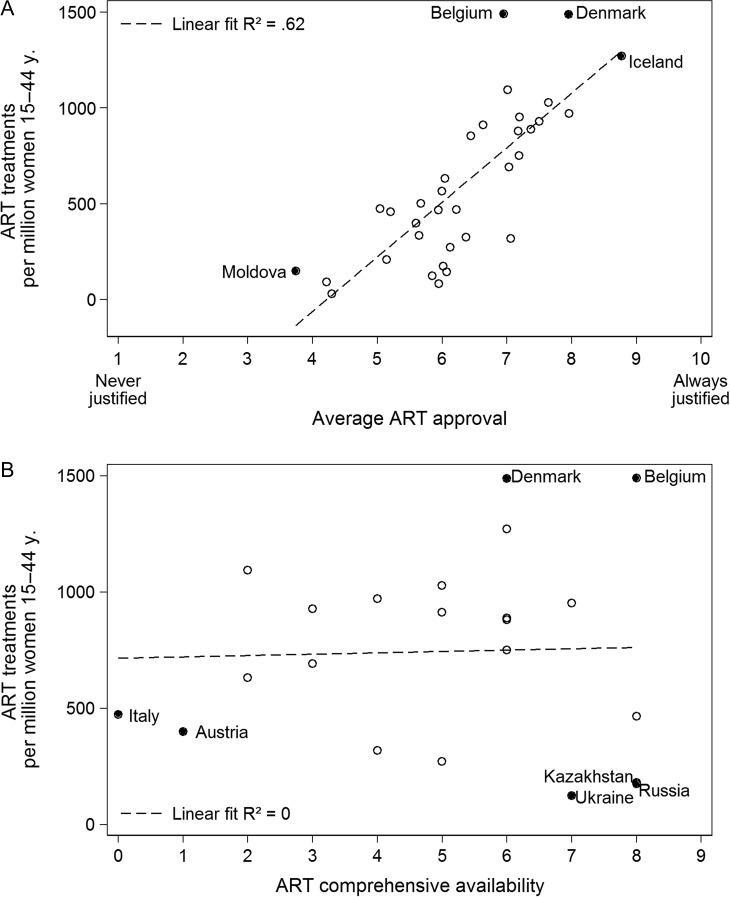
ART treatments by (**A**) average ART approval in a country, and (**B**) ART comprehensive availability, ca. 2010.

Figure 4A shows the correlation between average ART social approval in a country and ART treatments. We see a strong linear trend, meaning that the greater average ART normative approval in a country, the higher the number of ART treatments. The *R*-squared for ART approval is 0.62, indicating that 62% of the variance in ART usage can be explained by normative values, twice as high as GDP per capita. There is however no direct relationship between the comprehensive availability of ART as measured by our index and the actual number ART treatments, which can be seen in Fig. 4B (*R*-squared = 0.00). Some countries, such as Italy and Austria are highly restrictive in ART service availability and have relatively low ART usage, whereas other countries, such as the Ukraine and Kazakhstan have more comprehensive ART availability, yet still have low ART usage.

### Size of religious groups and type of religion

**Figure 5:**
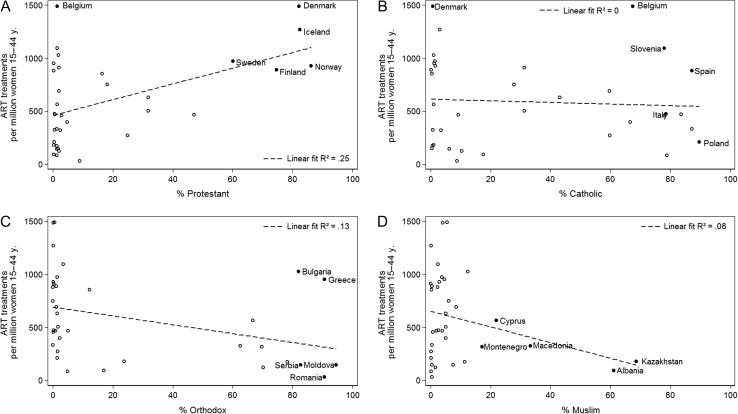
ART treatments by size of religious group: (**A**) Protestant, (**B**) Catholic, (**C**) Orthodox and (**D**) Muslim, ca. 2010.

Religion has also been shown as factor linked to ART usage (Benagiano, 2008; Adamson, 2009). Figure 5 shows the correlations between the size of religious Protestant, Catholic, Orthodox and Muslim groups in a country and the ART usage in this country. For the Protestant group (A), we find a sizable positive relationship with the number of ART treatments in a country (*R*-squared = 0.25) or, explained differently, a higher number of Protestants explains 25% of the variance in ART usage. Figure 5B reveals that—rather surprisingly, considering the doctrine of the Catholic Church in relation to ART—that there is no relationship between the size of the Catholic population in a country and the number of ART treatments. To illustrate this point, note that Poland, Italy, Spain and Slovenia are four countries with a high share (75%+) of Catholics, yet have very different levels of ART usage. We find negative correlations between the Orthodox (C) and the Muslim (D) population sizes and the number of ART procedures in these countries, with *R*-squares of 0.13 and 0.08, respectively. For the Muslim case, it is essential to acknowledge that there are only two Muslim-majority countries in our data, Albania and Kazakhstan, and thus results should be examined with caution.

### Multiple regression model

The results until now only show bivariate associations and lack an understanding of whether differences are statistically significant or differ when other factors are included together. Table I presents multiple OLS regression models, where predictor variables are entered in a stepwise fashion. OLS regression is a statistical approach that allows the examination of the relationship between an outcome variable, which in this case is ART usage, with one or several predictor variables. The coefficients obtained from a regression model can be interpreted as the average change in the outcome when a predictor variable increases by 1. In a regression model with multiple predictor variables, a coefficient can be interpreted as the average change in the outcome, holding all other predictors constant. Given the relatively low number of cases, we focus on those variables that have shown a substantial bivariate relationship in our analyses (Cohen *et al.*, 2003).

**Table I:** OLS regression models of ART usage, European countries, ca. 2010.

	(1)	(2)	(3)	(4)	(5)	(6)
GDP per capita (logged)	382.56^***^	279.22^*^	263.94^*^	99.02	−710.00	166.50
	[177.81,587.30]	[50.36,508.08]	[14.97,512.91]	[−92.44,290.48]	[−1426.45,6.45]	[−29.88,362.89]
% Protestants		4.41	4.27	−0.22	−3.59	−1.41
		[−0.49,9.31]	[−0.81,9.35]	[−4.28,3.84]	[−8.34,1.15]	[−5.49,2.67]
% Highly educated mid-aged women			7.83	−1.95	0.59	−68.81
			[−7.54,23.20]	[−13.74,9.85]	[−10.52,11.71]	[−140.85,3.24]
Avg. ART approval				276.03^***^	−1326.94	71.28
				[167.36,384.70]	[−2706.18,52.31]	[−170.07,312.63]
GDP per capita (logged) * Avg. ART approval					159.45^*^	
					[22.62,296.28]	
% Highly educated mid-aged women * Avg. ART approval						11.65
						[−0.75,24.06]
Constant	−3308.63^**^	−2331.14^*^	−2321.62	−2093.62^*^	5962.89	−1636.69
	[−5397.81,−1219.44]	[−4624.72,−37.56]	[−4807.35,164.11]	[−3896.45,−290.79]	[−1148.65,13074.43]	[−3425.22,151.84]
Observations	35	35	33	32	32	32
Adjusted *R*-squared	0.28	0.33	0.31	0.64	0.69	0.67
*F*-test	14.45	9.43	5.85	14.82	15.08	13.80
*df* Model	1	2	3	4	5	5
*df* Error	33	32	29	27	26	26

95% CI in brackets.

^*^P < 0.05, ^**^P < 0.01, ^***^P < 0.001.

Model (1) reiterates the finding already seen in Fig. 3A. A 1% increase in GDP is associated with 383 additional ART procedures per million women of reproductive age. Model (2) adds the percentage of Protestants to the equation, which reduces the coefficient between GDP and ART usage (to 279), yet is not significantly correlated with ART usage itself. The CI for the Protestant effect is arguably relatively wide (−0.49, 9.31), yet even the upper limit of the CI denotes a substantively unimportant effect: A one-percentage point larger Protestant population goes along with maximally nine additional ART treatments per million women of reproductive age. Model (3) adds the percentage of mid-aged women with tertiary education to the equation. The relationship between GDP and ART usage remains stable, and no statistically significant relationship between the percentage of mid-aged women with tertiary education and ART usage is found. Model (4) includes the average normative approval of ART in a country. The GDP coefficient reduces substantially in size (from 263.9 to 99.0) and becomes statistically insignificant (95% CI: −92.4, 290.5), yet the average ART approval coefficient is statistically significant and large: a one-point increase of average approval in a country associated with 276 additional ART treatments per million women of reproductive age.

As a robustness check of our findings, we have re-estimated our analysis replacing the percentage Protestants with the percentages of the other religious groups, and substantive findings remain the same. None of the religious group sizes are significantly related to ART usage in a country, and all of the other observed relationships hold (these models are shown in Supplementary Information, Table S1).

**Figure 6:**
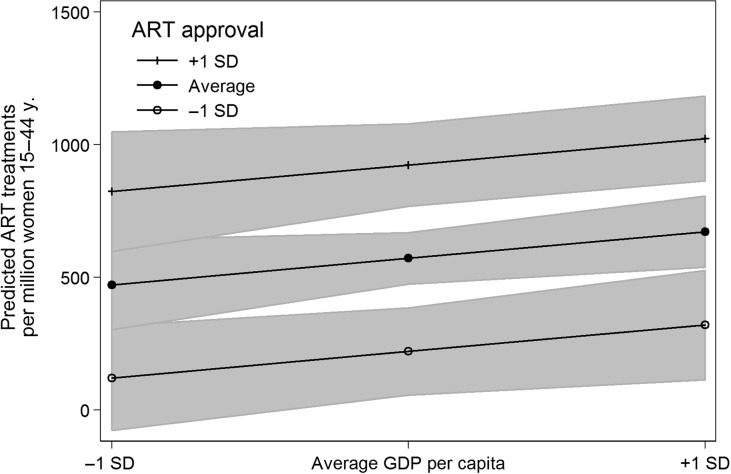
The positive relationship between ART approval and ART treatments is stronger at higher levels of GDP. Predictions based on Model (5) of Table I, error bands denote 95% CI.

But is it really only attitudinal factors that matter for explaining country differences in ART usage? Model (5) estimates a model that interacts GDP per capita and ART approval, testing the suspicion that ART approval really only is effective in increasing ART usage when a certain level of country wealth or in other words, demand and infrastructure, is available. Figure 6 illustrates this result by showing that the positive relationship between ART approval and the number of ART treatments is indeed stronger at higher levels of GDP. We did not find any significant interactions between ART approval and the share of Protestants or the share of highly educated, mid-aged women (Model 6).

## Discussion

This study linking financial, demographic, normative cultural, and religious composition of the population has generated a number of important insights. First, we were able to replicate the finding that country affluence is associated with the prevalence of ART treatments in a country. In other words, the wealthier a country, the greater the number of ART treatments.

Second, we showed that there are important non-economic factors also related to the number of ART treatments in our countries under study. Fertility postponement, measured by the size of biggest group of potential ART users for that reason, namely highly educated, mid-aged women (Barbuscia and Mills, 2017), is associated with ART usage only in a bivariate fashion. Religion is also correlated with ART utilization in a bivariate fashion; the share of Protestants is positively and the share of Muslims is negatively correlated with ART usage.

Thirdly, we were able to reveal surprising findings with respect to regulation and the share of Catholics in a country. According to our findings, making ART more accessible to more diverse groups and having more forms of ART available does not lead to a higher utilization of ART. It appears that the formal right to a certain form of treatment is less important than being provided the means to have such treatment. We speculate that the rights for access to many ART procedures may be irrelevant in the bigger picture, since ART is extremely expensive (Chambers *et al.*, 2014) and prohibitive on those grounds for most patients. With respect to the Catholic denomination, we showed that there is no relationship between the size of the Catholic population and ART usage in a country. This is counterintuitive given the position of the Catholic church (Congregation for the Doctrine of the Faith, 1987), but may be related to actual practicing Catholics and the disparity between religious prescriptions and translation into daily life.

The most striking implication of our findings is that ART research and policy-making should more openly acknowledge and work towards understanding the pivotal role that culture and normative values has on shaping assisted reproduction policies, accessibility, and usage (Präg *et al.*, 2017). Rather than focusing predominantly on the safety and efficacy of ART procedures, communication and education about the importance of access to ART and engaging in a wider public discourse appears to be an equally important policy strategy for increasing access and acceptance for ART. The 18th World Health Assembly recognized already in 1965 that ‘problems of human reproduction involve the family unit as well as society as a whole, and that the size of the family should be the free choice of each individual family’ (Eighteenth World Health Assembly, 1965, p. 35). This statement reinforced the rights of all to have children, and formulated an agenda for social policy to support those rights. Another finding of our study raises hopes in this respect. Despite the negative position of Catholic doctrines towards assisted reproduction, our analyses have shown that countries with a majority-Catholic population do not appear to be affected by this and often show average to high levels of ART usage.

One challenge that affects most research on ART in Europe is cross-border reproductive care. There is only limited quantitative data on the extent of ART recipients traveling to other countries (Shenfield *et al.*, 2010), and our conclusions are implicitly based on the assumption that all ART treatments are on the residents of their respective countries only. A central coding system that would allow ART recipients to be tracked across countries (De Geyter *et al.*, 2016) would thus not only be of great benefit to ART recipient care, but would also improve research on cross-national differences in ART usage. Another limitation of our analysis is that the economic factor we accounted for in our analyses was the affluence of a country, and not the out-of-pocket costs of treatment, which vary widely across countries (Chambers *et al.*, 2014) and are the costs that matters most to patients. We also need to acknowledge that our findings are based on an observational, cross-sectional study, making causal inference impossible. Although it is tempting to do so, our country-level results do not allow inferences to be made to the individual level, as this would constitute an ecological fallacy (Robinson, 2009). For instance, our country-level study shows that the share of Catholics in a population is unrelated to the number of ART treatments in that population. This does not mean that Catholics are just as likely to undergo ART treatments as individuals of another denomination; to come to such conclusions, the analysis of individual-level microdata is necessary.

## Supplementary data

Supplementary data are available at *Human Reproduction* online

## Supplementary Material

Supplementary Data

Supplementary Data
