# Supplementary material for: Cultural determinants influence assisted reproduction usage in Europe more than economic and demographic factors
Source: Hum Reprod. 2017 Sep 29;32(11):2305–14. doi: 10.1093/humrep/dex298 (PMC5850226; doi:10.1093/humrep/dex298)
Supplement: Supplementary Data [file dex298_supplementary_information.pdf]

Cultural Determinants Influence Assisted Reproduction Usage in Europe More than Economic and Demographic Factors: Supplementary Materials

Patrick Präg
University of Oxford and Nuffield College

Melinda C. Mills
University of Oxford and Nuffield College

Human Reproduction, https://doi.org/10.1093/humrep/dex298

Robustness check: using other religious groups as predictors

As mentioned in the article, we have re-estimated our analysis replacing the percentage Protestants with the percentages of the other religious groups (Table S1), and substantive findings remain the same. None of the religious group sizes are significantly related to ART usage in a country, and all of the other observed relationships hold.

Data used in our analyses

For quick reference, we are providing all data used in our analyses in Table S2.

Replication package

We provide readers with a full replication package to enable them to replicate our analyses (and conduct further analyses they might be interested in). The replication package to the article "Cultural Determinants Influence Assisted Reproduction Usage in Europe More than Economic and Demographic Factors" includes five files:

- replication.do, a Stata 13 command file which conducts the analyses and creates the tables and figures,

Table S1: OLS regression models of ART usage accounting for different religious groups, ca. 2010

	Protestants	Catholics	Orthodox	Muslims
GDP per capita (logged)	99.02	100.13	68.62	100.18
	[-92.44,290.48]	[-97.87,298.14]	[-173.55,310.79]	[-86.24,286.61]
Percentage religious group	-0.22	-0.16	-0.64	1.26
	[-4.28,3.84]	[-3.06,2.75]	[-4.27,2.99]	[-7.60,10.11]
Percentage Highly-educated mid-aged women	-1.95	-1.97	-1.24	-1.44
	[-13.74,9.85]	[-13.80,9.87]	[-13.52,11.04]	[-13.59,10.70]
Avg. ART approval	276.03***	271.81***	273.75***	274.42***
	[167.36,384.70]	[167.33,376.30]	[175.11,372.39]	[175.54,373.30]
Constant	-2093.62*	-2076.68*	-1771.17	-2116.10*
	[-3896.45,-290.79]	[-3780.68,-372.68]	[-4083.91,541.56]	[-3826.39,-405.81]
Observations	32	32	32	32
Adjusted R-squared	0.64	0.64	0.64	0.64
F-test	14.82	14.82	14.91	14.88
df model	4	4	4	4
df error	27	27	27	27

Notes: 95% confidence intervals in brackets, * $p < 0.05$, ** $p < 0.01$, *** $p < 0.001$

- data.csv, a comma-separated values file which includes the data being analyzed
- shapefile.shp, and
- shapefile.dbf, two files necessary to generate the maps in Figure **??**
- readme.txt, a text file containing the information given here.

To replicate the analyses, one needs to ensure that all four files are in the same directory, open replication.do in Stata and execute it.

Table S2: Data used in analyses, European countries, ca. 2010

Country	Abbr.	No. treatments	GDP per capita (logged)	% Educated women	ART Approval	Availability	Protestants	Catholics	Orthodox	Muslims
Albania	AL	92.6	9.26	0.8	4.2		0.3	17.6	17.1	61.1
Austria	AT	399.1	10.74	11.2	5.6	1	4.7	66.7	2.3	5.1
Belgium	BE	1490.2	10.65	22.9	6.9	8	1.4	67.5	0.6	5.5
Bulgaria	BG	1028.0	9.69	19.6	7.6	5	1.8	1.0	82.0	12.5
Croatia	HR	469.3	9.96	14.3	6.2		0.7	83.6	5.1	1.9
Cyprus	CY	565.9	10.37	24.7	6.0		1.4	1.0	66.8	21.9
Czech Republic	CZ	912.0	10.28	8.7	6.6	5	2.0	31.3	0.3	0.0
Denmark	DK	1488.0	10.71	24.5	8.0	6	82.2	0.7	0.2	4.1
Estonia	EE	854.5	10.17	31.4	6.4		16.4	0.4	12.2	0.3
Finland	FI	888.5	10.61	16.3	7.4	6	74.7	0.2	1.2	0.5
France	FR	692.4	10.54	11.5	7.0	3	2.0	59.7	1.2	8.6
Germany	DE	502.4	10.68	15.5	5.7		31.9	31.3	1.7	5.0
Greece	GR	952.6	10.14	31.9	7.2	7	0.2	1.2	90.6	4.6
Hungary	HU	271.8	10.04	20.0	6.1	5	24.9	59.8	1.5	0.2
Iceland	IS	1271.4	10.61	19.8	8.8	6	82.4	3.0	0.2	0.2
Ireland	IE	457.2	10.75	35.8	5.2		2.9	78.1	0.2	0.8
Italy	IT	473.9	10.50	10.0	5.0	0	0.5	78.7	0.2	2.6
Kazakhstan	KZ	179.7	10.02	19.1		8	0.3	1.0	23.8	68.6
Lithuania	LT	83.0	10.11	26.1	6.0		1.3	78.8	4.9	0.2
Macedonia	MK	325.9	9.38		6.4		0.5	0.7	62.6	33.4
Moldova	MD	147.8	8.35	11.9	3.7		1.7	0.5	94.5	0.4
Montenegro	ME	318.3	9.54		7.1	4	2.4	3.5	69.7	17.2
Norway	NO	928.9	11.09	21.5	7.5	3	86.2	1.7	0.1	2.9
Poland	PL	209.3	10.08	15.2	5.1		0.4	89.6	1.5	0.1
Portugal	PT	334.0	10.18	6.0	5.6		1.3	87.2	0.0	0.4
Romania	RO	30.2	9.85	8.6	4.3		8.9	8.9	90.6	0.5
Russia	RU	174.2	10.14	26.4	6.0	8	1.2	0.5	78.2	11.4
Serbia	RS	144.0	9.48	13.0	6.1		1.3	6.2	82.8	7.6
Slovenia	SI	1093.4	10.27	20.5	7.0	2	1.5	78.0	3.4	2.4
Spain	ES	880.2	10.37	20.7	7.2	6	0.3	87.2	0.0	2.3
Sweden	SE	971.8	10.71	24.5	8.0	4	60.1	1.5	1.4	3.8
Switzerland	CH	631.5	10.96	20.1	6.0	2	31.9	43.1	1.5	5.1
Netherlands	NL	750.6	10.75	19.4	7.2	6	18.2	27.8	0.0	6.2
Ukraine	UA	123.1	9.04	35.7	5.8	7	2.0	10.4	70.1	1.7
United Kingdom	GB	466.3	10.53	20.1	5.9	8	47.2	9.2	0.9	3.9
